# Bioinformatic Analyses and Integrated Machine Learning to Predict prognosis and therapeutic response Based on E3 Ligase-Related Genes in colon cancer

**DOI:** 10.7150/jca.98723

**Published:** 2024-08-19

**Authors:** Lunxi Liang, Xiao Liang, Xueke Yu, Wanting Xiang

**Affiliations:** 1 Department of Gastroenterology, The Affiliated Changsha Central Hospital, Hengyang Medical School, University of South China, Changsha, China.; 2 School of Clinical Medicine, Changsha Medical University, Changsha, China.; 3 Department of Pathology, The Affiliated Changsha Central Hospital, Hengyang Medical School, University of South China, Changsha, China.; 4 Department of Gastroenterology, The Third Xiangya Hospital, Central South University, Changsha, China.

**Keywords:** Colon cancer, Machine learning algorithm, E3 ubiquitin ligase, Immune response.

## Abstract

**Purpose:** Colorectal cancer is the third most common cause of cancer death worldwide. We probed the correlations between E3 ubiquitin ligase (E3)-related genes (ERGs) and colon cancer prognosis and immune responses.

**Methods:** Gene expression profiles and clinical data of patients with colon cancer were acquired from the TCGA, GTEx, GSE17537 and GSE29621 databases. ERGs were identified by coexpression analysis. WGCNA and differential expression analysis were subsequently conducted. Consensus clustering identified two molecular clusters. Differential analysis of the two clusters and Cox regression were then conducted. A prognostic model was constructed based on 10 machine learning algorithms and 92 algorithm combinations. The CIBERSORT, ssGSEA and TIMER algorithms were used to estimate immune infiltration. The OncoPredict algorithm and The Cancer Immunome Atlas (TCIA) predicted susceptibility to chemotherapeutic and targeted drugs and immunotherapy sensitivity. CCK-8, scratch-wound and RT‒PCR assays were subsequently conducted.

**Results:** Two ERG-associated clusters were identified. The prognosis and immune function of patients in cluster A were superior to those of patients in cluster B. We constructed a prognostic model with perfect predictive capability and validated it in internal and external colon cancer datasets. We discovered significant discrepancies in immune infiltration and immune checkpoints between different risk groups. The group with high-risk had a reduced half-maximal inhibitory concentration (IC50) for some routine antitumor drugs and reduced susceptibility to immunotherapy. *In vitro* experiments demonstrated that the ectopic expression of PRELP inhibited the migration and proliferation of CRC cells.

**Conclusions:** In summary, we identified novel molecular subtypes and developed a prognostic model, which will help a lot in the advancement of better forecasting and therapeutic approaches.

## Introduction

Colorectal cancer (CRC) is the third most frequent cause of cancer death in both females and males in the United States 1. With the rapid development of advances in early detection, targeted therapy and surgery in recent decades, progress in the treatment of tumors has accelerated, and there is a decreasing trend in the mortality of patients with colon cancer. However, early symptoms are not common in patients with colon cancer, and the disease is often confirmed at a very late stage, which leads to a low long-term survival rate because of limited treatment options 2. Consequently, given that colon cancer is a highly heterogeneous tumor 3, there is an urgent need for new strategies to predict the prognosis of colon cancer more credibly and guide individual treatment strategies. Importantly, recent studies indicate that risk models based on multigene expression are a viable option 4-6.

In colon cancer, the regulation of ubiquitination is an indispensable process that influences oncogene and tumor suppressor expression. Ubiquitination of the target protein is considered a three-step enzyme-linked reaction mediated by E1, E2, and E3 ubiquitin ligases. E3s are strongly responsible for substrate specificity 7. Not surprisingly, the activation of oncogenes and inactivation of oncosuppressors regulated by E3s have been implicated in the development of colon cancer. Proteasome-mediated degradation is important for the ubiquitylation involved in tumor development. For example, morn3 has been shown to accelerate the degradation of p53 by recruiting the E3 ligase MDM2 to promote the proliferation of colon cancer cells 8. Another famous tumor suppressor gene closely related to E3s is PTEN, which can be directly degraded by the E3 ligase WWP1 in colon adenocarcinoma 9. However, nonproteolytic ubiquitylation does not involve ubiquitin-dependent proteolysis but rather involves immunological signal transduction, protein-protein interactions, membrane trafficking, and chromatin regulation during carcinogenesis 10, 11. Wang and collegues 12 found that the ubiquitination of Sestrin2 by the E3 ligase RNF167 could influence its interaction with GATOR2 and further disturb mTORC1 signaling in colon cancer. Therefore, E3-related genes (ERGs) are considered promising targets. Furthermore, E3-based prognostic models have been proposed for bladder cancer 13, glioma 14, and hepatocellular carcinoma 15. Nonetheless, the value of ERGs in colon cancer classification and prognosis remains unknown.

Cancer progression is strongly influenced by the tumor microenvironment (TME), which facilitates patient prognosis and the identification of potential targets for tumor therapy 16. Ubiquitination is closely connected with the TME 17. Recently, T-cell-mediated cytotoxicity, which is most important in killing cancer cells, has been regarded as a possible way to avoid an immune response 18. PD-1 is a critical immune checkpoint on activated T cells and can promote immunosuppression. Ubiquitin-mediated modulation of the PD-1/PD-L1 pathway by E3s has obvious implications for immune responses in colon cancer 19. For example, the E3 ligase SPOP has been shown to be disrupted by aldehyde dehydrogenase 2 (ALDH2), thereby preventing the proteasome-dependent degradation of PD-L1, which consequently inhibits T-cell tumor infiltration in CRC 20. Nonetheless, the relationship between ERGs and the TME in colon cancer is not clear.

In the present study, for the first time, we focused on 240 E3s, conducted a holistic analysis of ERGs, identified ERG-associated molecular subtypes, identified a new prognostic model for colon cancer based on the integration of multiple machine learning algorithms, and elucidated the connections between the risk model and immune response as well as sensitivity to antitumor drugs. Finally, to reveal the role of PRELP, we conducted *in vitro* experiments and found that PRELP may function as a tumor suppressor in CRC. Our research provides novel perspectives on the underlying mechanisms and E3-associated targets involved in colon cancer and may also provide a foundation for individualized therapy.

## Materials and Methods

### Data collection

The transcriptomic and clinical information of normal colon samples and tumor samples was obtained from the Genotype-Tissue Expression (GTEx) database (https://www.gtexportal.org/) and The Cancer Genome Atlas (TCGA) database (https://portal.gdc.cancer.gov/). Additionally, we retrieved datasets for colon cancer from the GEO database, including the GSE17537 (n=54) and GSE29621 (n=65) datasets. The FPKM values of the RNA-seq datasets were log2 transformed. Ultimately, data from 430 tumor samples with survival information and 349 normal samples were combined for further study. All the data were extracted and annotated with Strawberry Perl and R software (version 4.4.0).

### Screening of E3 ubiquitin ligase (E3)-related genes (ERGs)

We retrieved 240 E3s from previously published literature. A list of those 240 E3s is provided in **Table S1.** Furthermore, the expression information of 240 E3s was integrated from the public database. On the basis of the expression information in the public database, coexpression analysis was performed using the "limma"package of R (version 4.3.1) in order to identify ERGs. Genes with p<0.001 and |R^2^| >0.4 were regarded as ERGs, which was refered to the previous literature 21.

### Analysis of differentially expressed ERGs between normal and cancerous tissues in the colon

By comparing the transcription profiles of samples in the GTEx and TCGA datasets with the “limma” R package, differentially expressed ERGs were identified (|log2-fold change (FC)| > 1, false discovery rate (FDR) < 0.01). Next, 1,493 ERGs with differential expression were analyzed for enrichment in GO (Gene Ontology) and KEGG (Kyoto Encyclopedia of Genes and Genomes) to investigate their biological functions and pathways (p < 0.05). Bubble, histogram, and circle plots were used for annotation analysis with the R packages “enrichplot”, “GOplot”, and “ggplot2” 22, respectively.

### Functional enrichment analysis by gene set enrichment analysis (GSEA)

The c2.cp.kegg.v7.5.1.symbols.gmt subclass was obtained from the Molecular Signatures Database (http://www.gsea-msigdb.org/gsea/downl-oads.jsp) for analyzing important pathways and molecular functions using specific gene expression profiles. To conduct the GSEA, we used the total transcriptome of tumor samples and considered gene sets with P < 0.001 and FDR, q < 0.001 as statistically significant.

### Weighted gene coexpression network analysis (WGCNA)

WGCNA 23, a freely accessible R software package, establishes a coexpression network by converting coexpression correlations into connection weights. Based on this background, ERGs were applied to establish a weighted gene coexpression network. The parameter β is a threshold parameter that emphasizes strong relationships and attenuates weak relationships between different genes. The weighted adjacency matrix was transformed into a topological overlap matrix (TOM) for assessing network connectivity, with hierarchical clustering used to create the clustering tree structure of the TOM. The genes were separated into different modules depending on different similarity measures based on the TOM. In this study, we set the mergeCutHeight, minModuleSize and deepSplit as 0.25, 60 and 2, respectively. Finally, we identified 11 coexpression modules. The eigengene dendrogram confirmed that in terms of the correlation coefficient, turquoise had the highest value of 0.93. A total of 788 hub genes in the turquoise module with module membership (MM) > 0.8 and gene significance (GS) > 0.5 were identified.

### Identification of ERG‑associated subtypes

409 potential ERGs were discovered by combining differentially expressed ERGs with hub genes from the turquoise module of WGCNA. Then, a Venn plot was used to display the intersection genes. Consensus clustering was applied to identify distinct E3-associated classifications by the k-means algorithm based on the 409 ERGs by using the “ConsensusClusterPlus” R package 24. The Kaplan-Meier method was used to evaluate overall survival (OS) between the two clusters. Furthermore, immune genes, immune cells and immune functions were compared. Finally, a total of 800 DEGs (|logFC|>1, p<0.05) between the two ERG‑associated clusters were identified using the R package “limma” 25.

### Risk model constructed based on integrated machine learning approaches

A high-accuracy risk model was constructed using ten machine learning algorithms and 92 algorithm combinations. The 10 algorithms used for integration included Lasso, Ridge, random survival forest (RSF), stepwise Cox, generalized boosted regression modeling (GBM), CoxBoost, supervised principal component (SuperPC), survival support vector machine (survival-SVM), elastic network (Enet), and partial least squares regression for Cox (plsRcox). The specific steps are described by Liu 26.

### Validation of the risk model

Randomly, patients were segmented into training and testing datasets at a 1:1 ratio by employing the “caret” R package. Using the "survminer" package, an optimal cutoff point was calculated. Then, the training and validation datasets were segmented into high- and low-risk patients. The risk model's ability to predict was shown through survival analysis, risk plots, and receiver operating characteristic (ROC) curves in the TCGA training dataset. R package “timeROC” was used to predict overall survival. Furthermore, the prognostic model was validated in a validation dataset (TCGA testing dataset, TCGA entire dataset, GSE17537 and GSE29621). Afterward, different risk subcategories and clinical characteristics, such as age, gender, TNM stage, tumor size, metastasis status, and lymph node involvement, were examined within the entire TCGA database. In addition, we employed univariate and multivariate Cox regressions to confirm these autonomous prognostic indicators. Finally, a prognostic nomogram was developed, followed by the utilization of the “rms” R package to create a calibration plot using the coefficients.

### Comparison of immune infiltration and immune characteristics among different risk subgroups

We applied different algorithms, including ESTIMATE, CIBERSORT and single-sample gene set enrichment analysis (ssGSEA), to evaluate immune cell abundance and immune function in different risk groups of patients with colon cancer. The R package “ESTIMATE” 27 was applied to estimate the three immunoscores between different risk groups: the stromal, immune, and ESTIMATE scores. Moreover, we used the CIBERSORT 28 method with the expression profiles of patients with colon cancer to evaluate the infiltration of 22 types of immune cells in the high- and low-risk subclasses. Furthermore, ssGSEA 29 was applied to explore the enrichment of immune functions, and the correlations between these functions and risk scores were subsequently determined by Pearson correlation analysis Which was finished by using “GSVA” package of R software 30. Additionally, we explored the connections between immune checkpoints and different risk groups as well as the 12 risk genes.

### Comparison of immune checkpoints and sensitivity to routine chemotherapy drugs in the two risk subgroups

Initially, we predicted the effectiveness of immunotherapy by applying the Tumor Immune Dysfunction and Exclusion (TIDE) algorithm 31. Drug resistance is an obstacle to cancer treatment. Thus, to explore the sensitivity of routine chemotherapy drugs in the two subgroups, we computed and compared the half maximal inhibitory concentration (IC50) values of different antitumor drugs by employing the Wilcoxon signed-rank test with the R packages “ggplot2” and “oncoPredict” 32.

### Mutation characteristics of the two risk subgroups

To elucidate the correlations between gene mutations and the two risk subgroups, we downloaded mutation information associated with colon cancer from the TCGA database. Then, the somatic variants in the two risk subgroups were comprehensively explored using the “Maftools” R package 33.

### Cell culture, plasmid transfection and antibodies

RPMI 1640 medium containing 10% fetal bovine serum was used to culture the Hct-116 and Lovo colorectal cell lines at 37 °C in a humidified environment with 5% CO_2_. The PRELP-FLAG gene was produced by GENECHEM (Shanghai, China) and transferred to colorectal cells by Lipofectamine 3000 (Thermo Fisher Scientific, #L3000150). Standard procedures were followed to maintain stable infection. FLAG-tag antibodies (Cell Signaling Technology, #8146) were used to detection the expresson of PRELP in this study.

### Quantitative real-time PCR (qRT‒PCR)

The mRNAs were isolated using TRIzol Up (TransGen Biotech, #ET111-01-V2) and reverse-transcribed into single-strand cDNA using a reverse transcription kit (TransGen Biotech**,** #AT341-01). For RT‒PCR, PerfectStart® Green qPCR SuperMix (TransGen Biotech, #AQ601-01-V2) and PCR primers (PRELP: forward, 5'-GAACCAGCAGAGCCAACAGACC-3', and reverse, 5'-CAGGTTGCGGCTATCACAGTAGAG-3') were used in a LightCycler®96 Real-Time PCR machine (Roche). The instructions provided with the reagents were used to perform the test.

### Western blotting

Proteins were lysed using RIPA lysis buffer (Beyotime Biotechnology, #P0013B). Protein levels were quantified using the BCA protein assay kit (Thermo Fisher Scientific, #23225). After boiled with 5× protein loading buffer and separated using 10% SDS-PAGE, the denatured protein was then transferred onto a PVDF membrane. 5% skimmed milk was used to block non-specific binding sites for 1.5 h at room temperature. We put the protein bands into box and make it react with the primary antibody overnight at 4 °C. Following three washes with TBST for 10 min, the protein bands was exposed to second HRP-conjugated antibodies for 1 h at room temperature. The membrane was subjected to blotting using a chemiluminescence system (BIO-RAD, USA).

### Cell Counting Kit-8 (CCK8) and scratch-wound assays

A CCK8 assay (Beyotime Biotechnology, #C0037) was conducted to determine cell viability. Briefly, 100 µl of culture medium containing 3.0×10^3 cells was added to 96-well plates to determine cell viability. Then, 10 µl of CCK8 solution was added to each well at the indicated time points, and the absorbance at 450 nm was measured. For the scratch-wound assays, 1×10^6 colorectal cancer cells were inoculated in 6-well plates containing culture medium. A 200 µl pipette tip was used to create scratches after the cells reached 90% confluency. The cells were washed and then cultivated in serum-free RPMI 1640 medium, after which the medium was discarded. After wounding, the cells were photographed 0 and 48 hours later. We calculated the wound closure area as follows: migration area (%) = (M0 - Mn)/M0×100. In this case, A0 is the initial wound area, and An is the remaining wound area at the metering point.

### Statistical analysis

We used R version 4.4.0 to analyze the clinical and expression profile information. Survival analysis was implemented using Kaplan-Meier curves and the Wilcoxon log-rank test. Two independent samples were compared using either the Mann-Whitney U test or t test. Categorical data were analyzed with Fisher's exact probability method or the χ2 test. A P value less than 0.05 was deemed statistically significant, with ns representing not significant, * representing P less than 0.05, ** representing P less than 0.01, and *** representing P less than 0.001.

## Results

### Identification of ERGs

An overview of this research can be found in **Figure S1**. Initially, 240 E3s were identified from previous literature (**Table S1**). After acquiring the data from the TCGA-COAD and GTEx datasets, we integrated the expression information of 240 E3s. To identify ERGs, we used coexpression analysis. Then, based on these E3s, we applied Spearman correlation analysis to identify ERGs in colon cancer samples. Finally, a total of 6,268 ERGs were selected (|R^2^|>0.4 and p < 0.001).

### ERGs that were expressed differently in colon cancer and normal tissues

**Figure 1:**
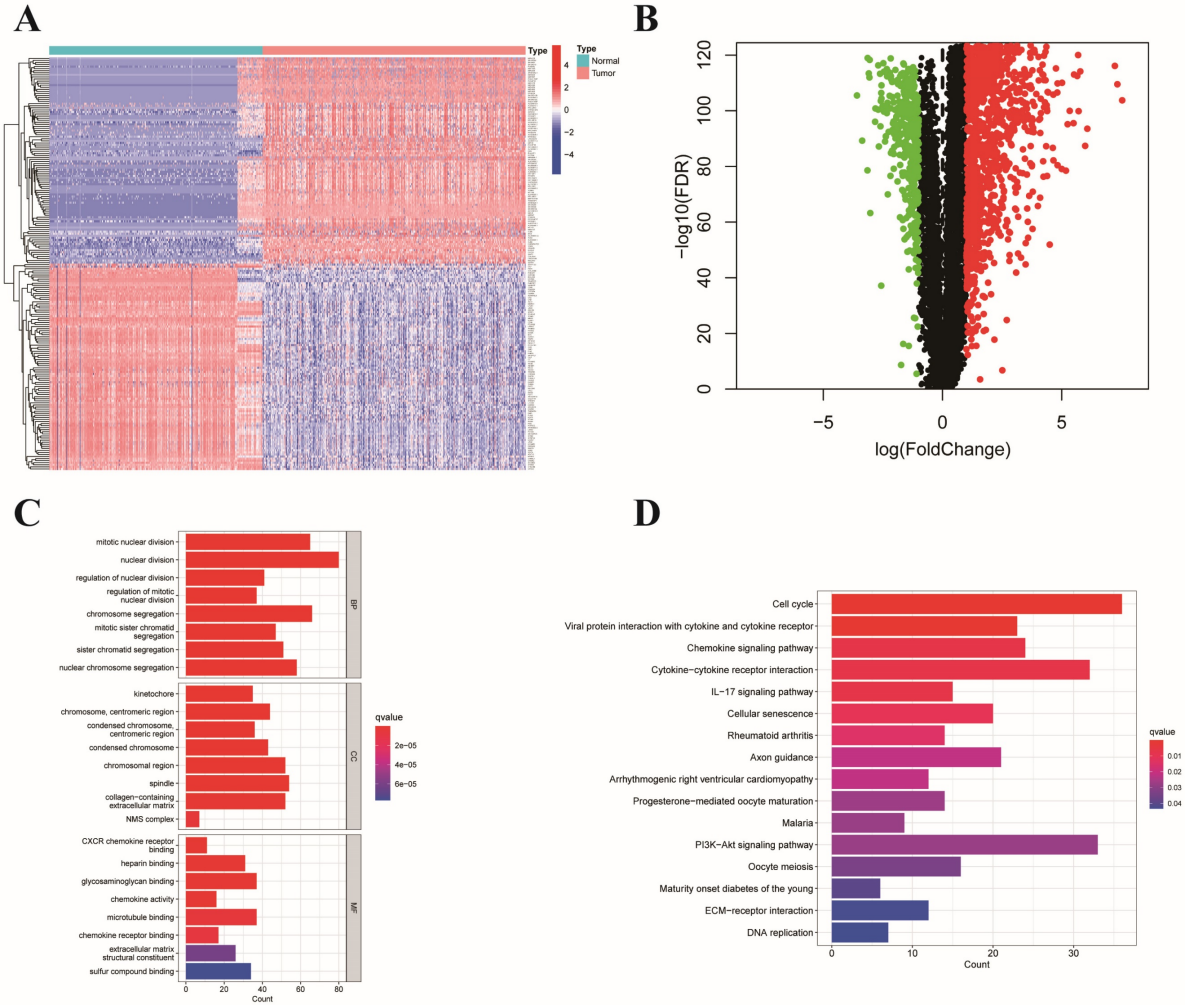
** Presentation and functional annotation of differentially expressed ERGs.** (A) Heatmap showing fifty differentially expressed ERGs, with red dots indicating significantly upregulated genes and blue dots indicating significantly downregulated genes; (B) Volcano map of differentially expressed ERGs between colon tissues and normal tissues; Histogram depiction (C) in the aspects of the BP, CC, MF categories; Histogram depiction (D) of the top 16 enriched pathways.

We identified 1,493 differentially expressed ERGs in colon cancer samples compared to normal samples from the TCGA and GTEx datasets. Among these genes, 1,117 were upregulated, and 376 were downregulated. A heatmap depicting 50 genes, including upregulated and downregulated genes, is shown in **Figure 1A**. In addition, a volcano plot of the differentially expressed ERGs is presented in **Figure 1B**. GO analysis revealed that the differentially expressed genes were related to mitotic nuclear division, nuclear division, and chromosome segregation in the biological process (BP) category. Regarding the molecular function (MF) category, differentially expressed genes were associated with CXCR chemokine receptor binding, microtubule binding, and glycosaminoglycan binding. Moreover, they were principally enriched in collagen-containing extracellular matrix, chromosomal region, and spindle in the cellular component (CC) category (**Figure 1C**). According to the KEGG analysis, genes related to cytokine-cytokine receptor interactions, the cell cycle, and the IL-17 signaling pathway were enriched (**Figure 1D**).

### Identification of hub genes and critical modules with WGCNA

**Figure 2:**
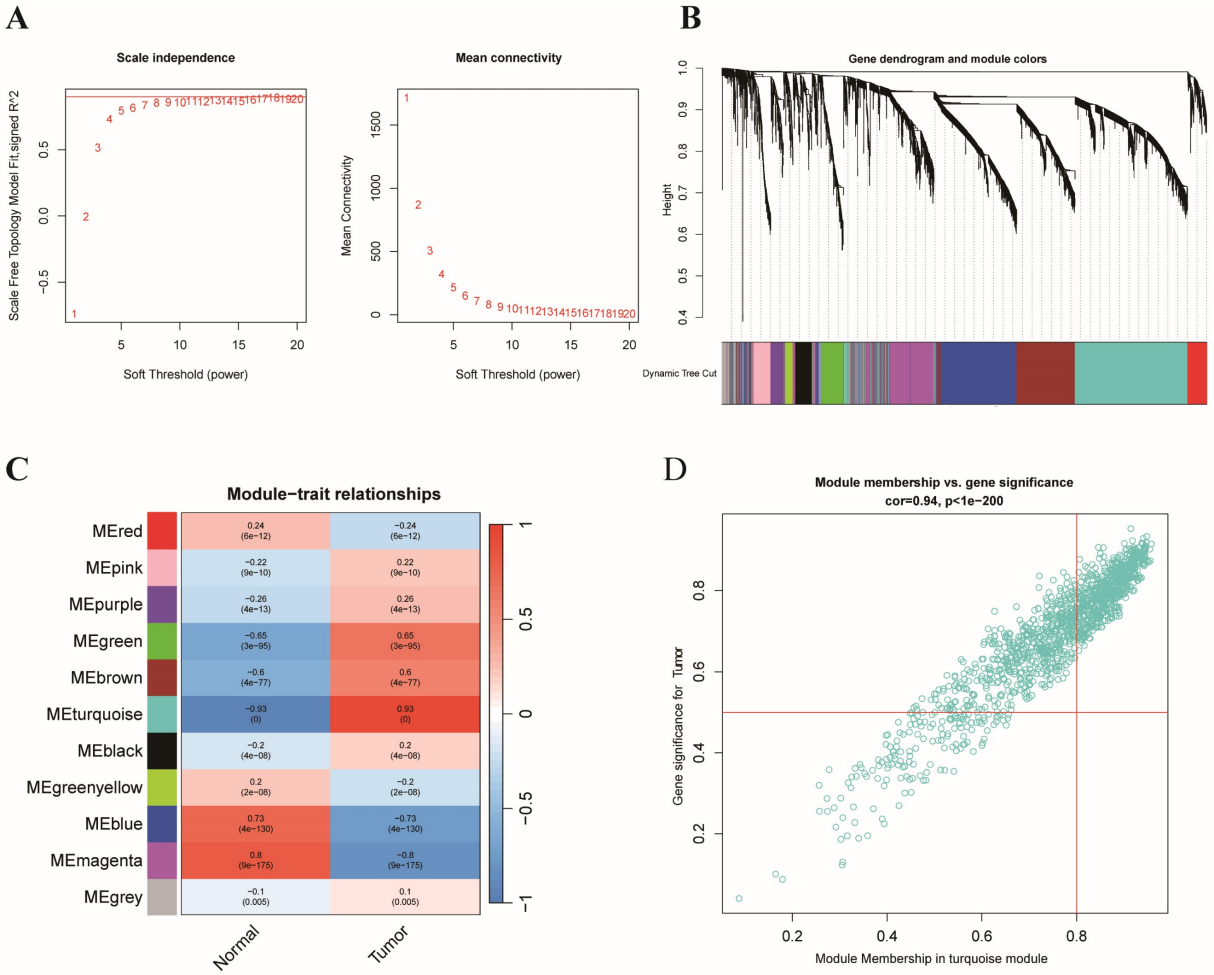
** WGCNA of ERGs and identification of E3-related hub genes.** (A) Analysis of the scale-free index and mean connectivity for the confirmation of soft-thresholding powers; (B) Hierarchical clustering dendrogram of ERGs in a variety of modules; (C) Correlation analysis between different tissues and the module eigengenes; (D) Scatter plots of GS score and MM for ERGs in the turquoise module.

On the basis of the expression matrix of 6,268 ERGs, we established a weighted gene coexpression network. Samples from the TCGA and GTEx datasets were separated into tumor samples (430 samples) and normal samples (349 samples). In **Figure 2A**, the horizontal axis represents the threshold, and the vertical axis represents the evaluation parameters of the scale-free networks. As the network's evaluation parameters increase, they become more consistent with the characteristics of a scale-free network. The horizontal line in the graph indicates a threshold value of 0.90. To create a scale-free network (**Figure 2A**), we established a soft threshold power β of 8 based on the connection between the soft threshold and mean connectivity. The ERGs with analogous expression types converged into the same modules with a cutting height difference of less than 0.25, which ultimately produced 11 coexpression modules (**Figure 2B**). The results revealed that 788 genes in the turquoise module (cor=0.93, p=0.000) were strongly associated with colon cancer (**Figure 2C-D**). In brief, the findings illustrated that the turquoise module was closely associated with colon cancer. Therefore, 788 hub genes of the turquoise module were subjected to further investigation.

### Identification of prognostic clusters based on ERGs in patients with colon cancer

**Figure 3:**
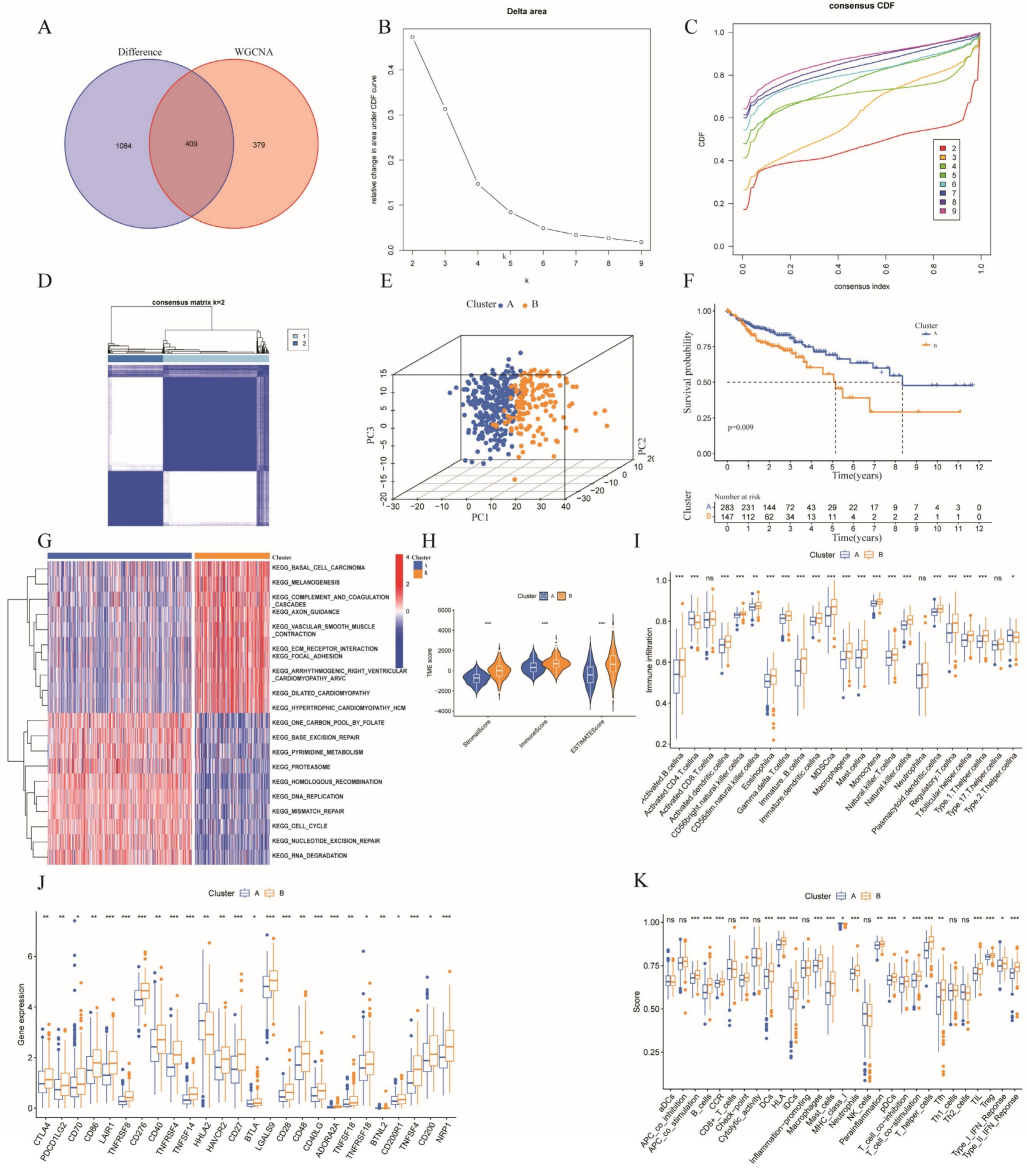
**Consensus clustering.** (A) Venn diagram analysis showing the overlapping ERGs of the differentially expressed ERGs and hub genes from WGCNA; (B) The relative change in the area under the CDF curve from k = 2 to 9;(C) Empirical CDF plots from 2 to 9; (D) Consensus matrix heatmap for K = 2; (E) PCA analysis based on the two clusters; (F) Kaplan-Meier curves of OS in the two clusters in colon cancer; (G) GSVA representing the different biological pathways between the two clusters;(H) Correlation between two clusters and the tumor microenvironment;(I)Comparison of immune-related cells between cluster A and B;(J) Comparison of immune checkpoints between cluster A and B;(K) Comparison of immune functions between cluster A and B.

By identifying the overlapping genes among the hub genes of the turquoise module with WGCNA and the differentially expressed ERGs, we identified 409 overlapping genes (**Figure 3A**). Among the 409 overlapping ERGs evaluated, we identified distinct prognostic subtypes by using consensus clustering, which revealed that the best number of clusters was two. Specifically, the delta area of the cumulative distribution function (CDF) showed that two was the most suitable number of clusters (**Figure 3B-D**). In addition, clusters A and B were distinct according to a principal component analysis (PCA) plot (**Figure 3E**). Based on the findings from Kaplan-Meier survival analysis (**Figure 3F**), samples in cluster B had a worse prognosis than those in cluster A. According to GSVA, cluster A was enriched in pathways associated with DNA repair (base excision repair, nucleotide excision repair) and the cell cycle, while cluster B was related to ECM-receptor interaction, basal cell carcinoma, and focal adhesion (**Figure 3G**).

To further understand the correlation between the two clusters and immunity, the "ESTIMATE" package was used to evaluate the TME scores (immune score, stromal score, and ESTIMATE score) of the two clusters. Regarding the TME score, higher immune scores or stromal scores indicate greater relative abundances of immunocytes or stromal cells in the TME, and the ESTIMATE score represents the aggregation of immune scores and stromal cells in the TME. The results revealed higher TME scores in patients in cluster B (**Figure 3H**). In addition, the numbers of immune cells in these two clusters are compared in **Figure 3I**, which illustrates a significant discrepancy in the immune response between the two clusters. Moreover, comparisons of immune scores revealed that patients in cluster B possessed stronger immune functions than patients in cluster A, while the tumor purity was greater than that of patients in cluster A (**Figure 3K**). Furthermore, we compared the expression levels of immune checkpoints in the two clusters and demonstrated that most checkpoints, such as CTAL4, were more strongly expressed in cluster B (**Figure 3J**). Additionally, combining with the results of GSVA, cluster B had significantly more pathways promoting tumor development than did cluster A. Collectively, the findings provide a basis for supporting the greater degree of malignancy of cluster B. Immune infiltration analysis revealed that cluster B had higher immune scores than did cluster A, which may be partially explained by intra- and intertumor heterogeneity and the presence of many exhausted immune cells in the TME 34.

### Construction of an ERGs signature associated with prognosis

To further study the potential mechanisms of these two tumor clusters, we obtained 800 DEGs between clusters A and B. Moreover, we screened 62 genes connected with OS in patients with colon cancer by univariate Cox regression analysis (**Table S2**). Then, we randomly divided the colon cancer samples into training and testing datasets at a 1:1 ratio. Afterward, a prognostic model was constructed based on 10 machine learning algorithms and 92 algorithm combinations.

**Figure 4:**
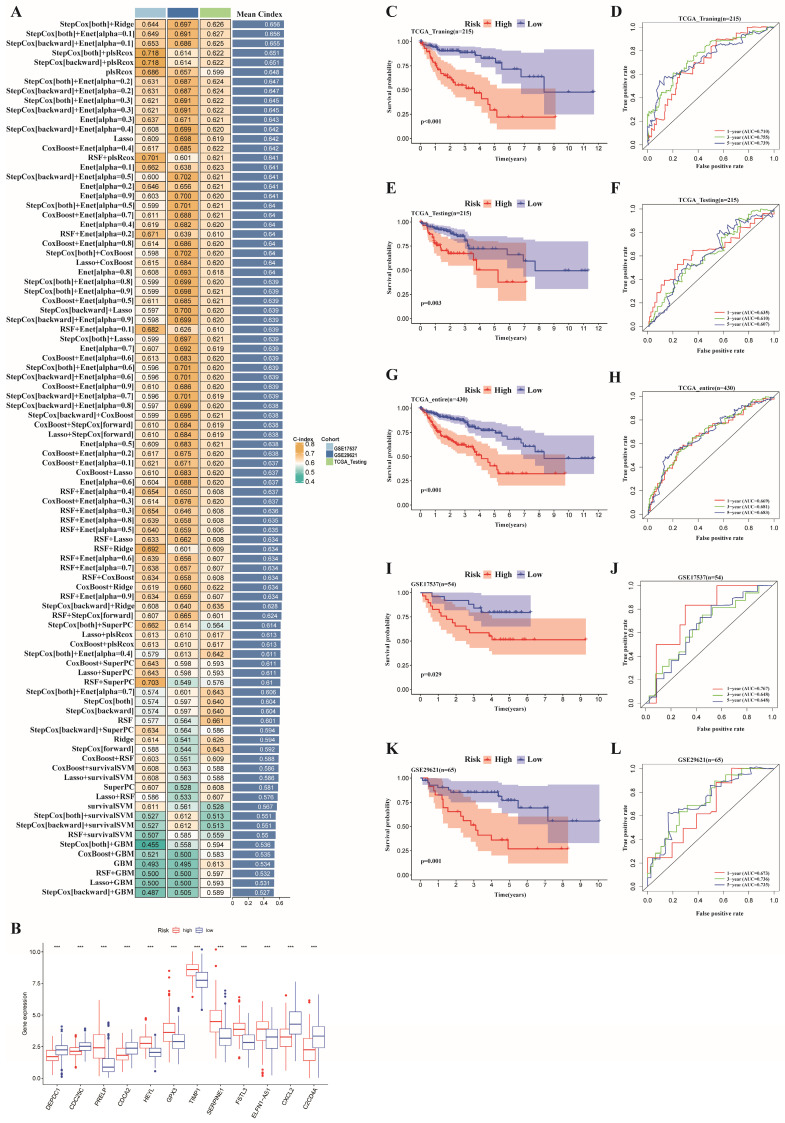
A prognostic model was constructed and validated based on 10 machine learning algorithms and 92 algorithm combinations**.** (A) A total of 92 kinds of prognostic models though LOOCV framework and the C-index of each model in all validation datasets; (B) Differential expression of the 12 risk genes between high- and low- risk subgroup; (C,E,G,I,K)Kaplan-Meier survival curves revealed that patients in the high-risk group had a worse prognosis compared with those in low-risk group in the training and validation dataset. (D,F,H,J,L) ROC curves validated the prognostic capability of the risk model based on risk genes in the training and validation dataset.

In the TCGA training dataset, 92 kinds of forecasting models were fitted through the LOOCV framework. Additionally, C-indexes were calculated for each model, including the TCGA testing dataset and the GSE17537 and GSE29621 datasets (**Table S3**). Ultimately, the two best risk models for all the validation datasets with the highest average C-index (0.656) were identified (**Figure 4A**). The first one was a combination of stepwise Cox (direction = both) and Ridge consisting of 24 genes, while the other was a combination of Cox (direction = both) and Enet (alpha=0.1) consisting of 12 genes (**Table S3**). Notably, the 12 genes comprising the latter model were included in former model. Therefore, we chose the latter model constructed by the combination of Cox (direction = both) and Enet (alpha=0.1) for further study. The 12 genes in the optimal risk model were DEPDC1, CDC25C, PRELP, CDCA2, HEYL, GPX3, TIMP1, SERPINE1, FSTL3, ELFN1-AS1, CXCL2, and C2CD4A.

The expression levels of the 12 risk genes above significantly differed between the two risk groups (**Figure 4B**). Furthermore, we applied the risk scores of the optimal model obtained from machine learning algorithms to classify all the individuals as high- or low-risk on the basis of optimal cutoff point value of training dataset. Furthermore, patients in the low-risk subgroup had obviously better overall survival (OS) in the TCGA training dataset (p < 0.001) (**Figure 4C**). Additionally, an ROC curve was created to confirm the precision of the training data, showing AUC values of 0.710, 0.755, and 0.739 for 1-, 3-, and 5-year OS, respectively (**Figure 4D**). The expression of the risk genes in different risk subgroups of patients with colon cancer, as well as the increased risk scores accompanied by increasing patient mortality, are revealed in the heatmap in **Figure S2A-B**.

### Evaluation of the ERGs-related risk model

On the basis of the optimal cutoff point of the risk score, the patients in the validation dataset were divided into low- and high-risk subgroups. We further explored the TCGA testing dataset and the entire dataset as well as two GEO datasets (GSE17537 and GSE29621) as validation datasets to verify the reliability and accuracy of prognosis prediction. The validation set showed that patients in the low-risk group had a higher OS rate compared to patients in the high-risk group according to Kaplan-Meier survival analysis (**Figure 4E, 4G, 4I, 4K**). The AUCs for 1-, 3-, and 5-year OS in the TCGA testing dataset, the entire TCGA dataset, GSE17537, and GSE29621 were 0.635, 0.610, and 0.607; 0.669, 0.681, and 0.683; 0.768, 0.648, and 0.648; and 0.673, 0.736, and 0.735, respectively (**Figure 4F, 4H, 4J, 4L**). In addition, the risk score, risk plot, and risk gene expression in the validation dataset (**Figure S2C-J**) corresponded to those in the training dataset.

**Figure 5:**
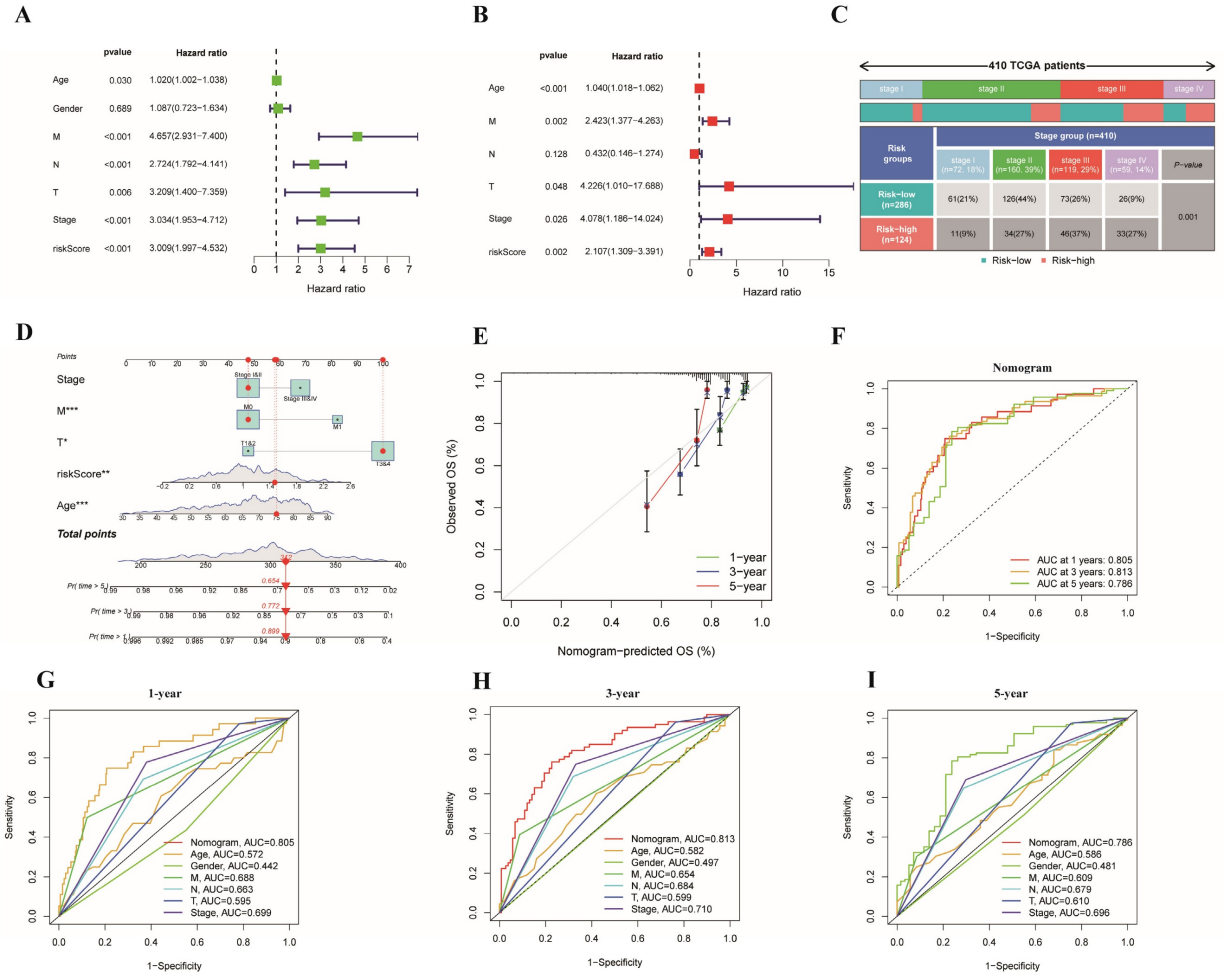
** Cox regression analyses of OS and the establishment of a nomogram model.** (A) Forest plot of univariate regression analyses; (B) Forest plot of multivariate Cox regression to identify the independent prognostic factors; (C) Distribution of tumor stage in the high- and low-risk groups in the TCGA cohort (p=0.001);(D) A nomogram model integrating the risk score and clinical features for the prediction of OS in colon cancer patients in the whole TCGA dataset; (E) Calibration plots of 1-, 3- and 5-year OS probabilities of the nomogram model; (F-I) AUC curves of 1-, 3-, and 5-year OS verified the potential prediction value of the prognostic indicators.

As the prognostic risk model was verified well in the validation set, we used the whole TCGA dataset for further study. In addition, we considered clinical characteristics (such as age, sex, tumor stage, T stage, M stage, and N stage) to identify whether the risk model could be applied as the best predictive factor for OS. Then, univariate and multivariate Cox regression analyses were conducted to determine whether the ERG-related risk score could be considered an independent predictor. Univariate Cox analysis revealed that age (p=0.030), tumor stage (p< 0.001), T stage (p= 0.006), M stage (p< 0.001), N stage (p< 0.001) and risk score (p < 0.001) were associated with prognosis (**Figure 5A**), while multivariate Cox regression analysis revealed that age (p< 0.001), tumor stage (p < 0.05), T stage (p= 0.048), M stage (p=0.026) and risk score (p < 0.001) may be independent prognostic markers for colon cancer (**Figure 5B**). In addition, stage II was the main constitution in the low-risk group, whereas stage III accounted for a large proportion of the high-risk group (**Figure 5C**). Tumor stage exhibited notably discrepancy between the two risk subgroups. Finally, considering the clinical features and gene expression, we integrated the risk model based on 12 risk genes, age, T stage, M stage and tumor stage into a prognostic nomogram, which could be applied to forecast the OS of patients with colon cancer in terms of 1-, 3-, and 5-year OS (**Figure 5D**). Moreover, a calibration plot for 1-, 3- and 5-year OS probabilities revealed reasonable consistency between the nomogram predictions and the actual observations (**Figure 5E**). In addition, we explored the AUC values for 1-, 3-, and 5-year prognosis in the entire cohort and found that the prognostic nomogram model had the optimum AUC values among those factors (**Figure 5F-I**).

### Clinical correlation and stratified analyses of different risk groups

**Figure 6:**
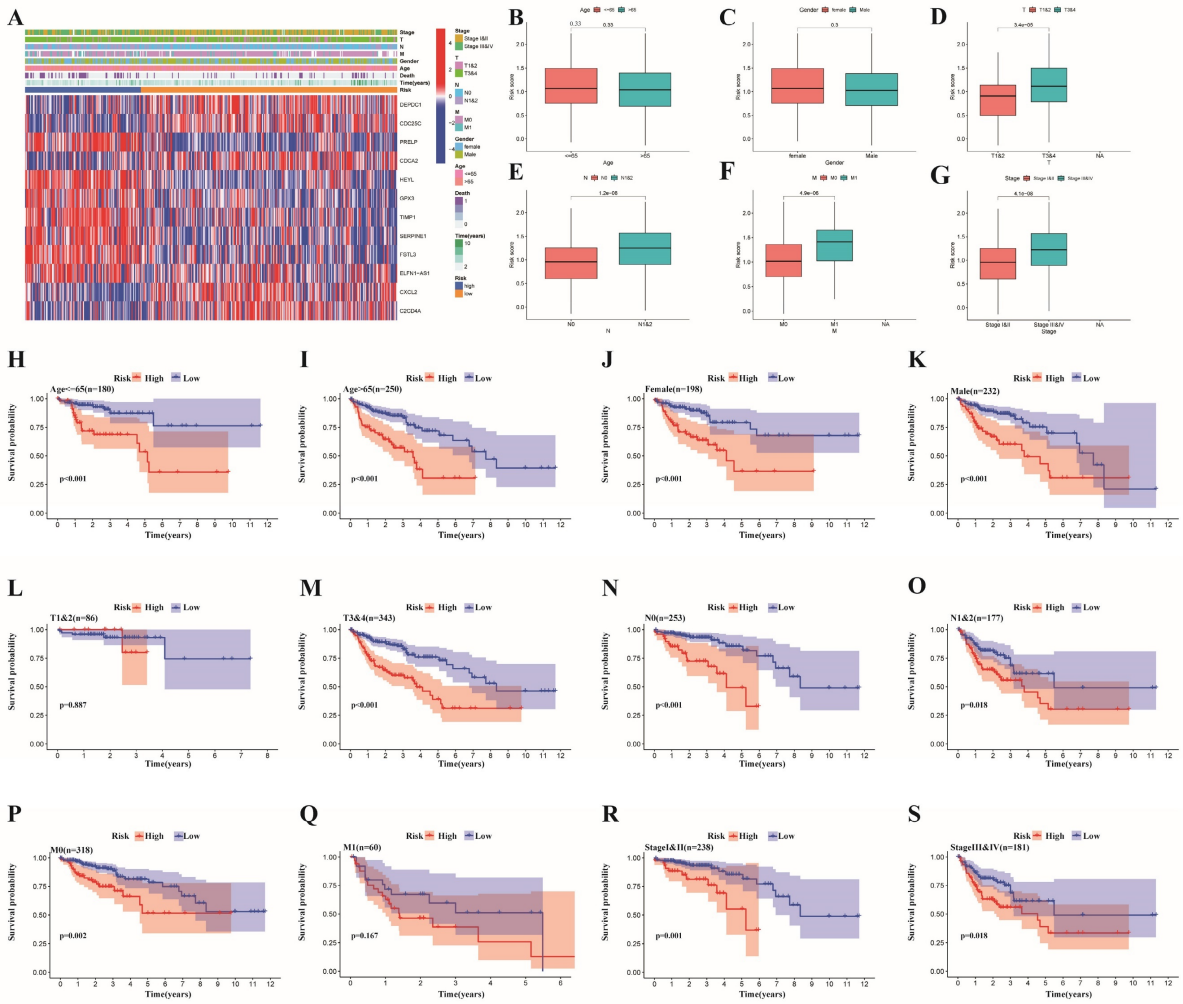
**Clinical correlation analysis and stratified analysis of the high- and low-risk subgroups.** (A) A heatmap of the correlations between clinical features and risk subgroups, as well as risk genes;(B-G) A strip chart revealed the difference of clinicopathological features between different risk subgroups(***p < 0.001; **p < 0.01;*p < 0.05); Kaplan-Meier curves showing the prognostic value of different risk subgroups for the patients separated by each clinicopathological feature. (H,I) Age; (J,K) Gender; (L,M) T Stage; (N,O) N stage; (P,Q) M stage; and (R,S) stage.

We divided some clinical features into 2 subgroups, and the correlations between clinical features and risk subgroups, as well as risk genes, are shown in the heatmap in **Figure 6A**. Specifically, we found that age, TNM stage, T stage, N stage and M stage were obviously different between the two risk groups (**Figure 6B-G, Table S4**). Furthermore, a stratified analysis of the two subgroups was performed. The risk score could be used to markedly stratify colon cancer patients into low- and high-risk groups on the basis of different clinical characteristics (such as age >65 years, age ≤65 years, male and female sex, stages I&II, stages III&IV, T3&4, N0, N1&2 and M0), illustrating the model's excellent predictive ability based on a variety of clinical features (**Figure 6H-K, 6M-P, 6R-S**). According to the findings from the K-M analysis, the risk model had obviously different risk stratification properties in patients with colon cancer, suggesting that patients with low-risk colon cancer had an obviously better prognosis. However, this conclusion was not reached for the T1-2 (**Figure 6L**) or M1 (**Figure 6Q**) subgroups.

### Discrepancies in immune infiltration, levels of immune checkpoints and somatic mutation characteristics between the two risk subgroups

**Figure 7:**
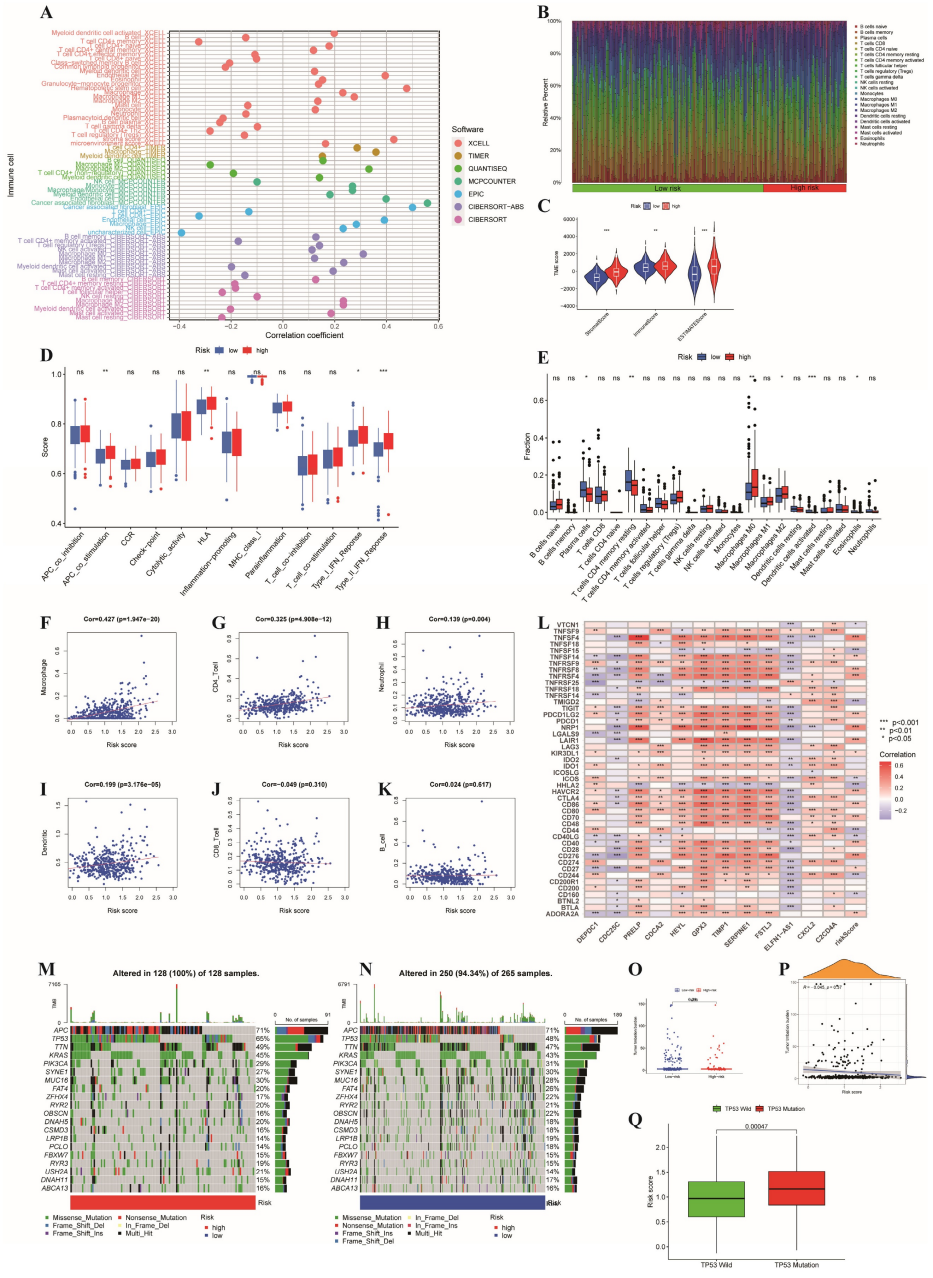
** Relationships between the risk signature and immune infiltration, immune checkpoint genes and somatic mutation features.** (A) Correlation between risk scores and immune cell abundance based on different software programs;(B) The proportion of 22 immune cells between the high- and low-risk subgroups;(C) The discrepancy of tumor microenvironment between high- and low-risk subgroups; (D) Comparison of immune functions between the different risk groups; (E) Comparison of immune cell between the different risk groups;(F-K) Pearson correlation analysis between the risk score and infiltration of six types of immune cells; (L) Correlations between immune checkpoints and risk scores as well as risk genes; (M,N) Waterfall plot of somatic mutation characteristics in the high- and low-risk subgroups; (O,P) The relationships between the TMB and risk signatures; (Q) Correlation analysis between risk scores and the mutated gene(TP53). ***p < 0.001; **p < 0.01; *p < 0.05.

The whole TCGA dataset was used to determine the associations between the prognostic risk model and immune cell abundance, immune checkpoint molecule levels, and therapeutic effects. The advancement of colon cancer is impacted by immune cells infiltrating the tumor microenvironment. Initially, we found that there was a close relationship between risk score and immune cell abundance based on different software programs (**Figure 7A**). In addition, the proportions of immune cells in the two risk groups are shown in **Figure 7B**, which illustrates a significant discrepancy in the immune response between the two risk subgroups. Additionally, we evaluated the TME scores of the two risk subgroups. The TME scores of patients were greater in the high-risk group than in the low-risk group (**Figure 7C**). In the high-risk subgroup, we revealed that M0 and M2 macrophages were more common, while in the low-risk subgroup, plasma cells, eosinophils and CD4 memory resting T cells were more abundant (**Figure 7E**). Furthermore, we investigated the correlation between the risk score and the infiltration of 6 types of immune cells and demonstrated that the risk score had the strongest correlation with macrophages (p<0.001, cor>0.4) (**Figure 7F-K**). The M1 and M2 subgroups are derived from M0 macrophages. It has been widely reported that M2 macrophages can facilitate tumor development 35. Previous study has demonstrated that the invasion of resting CD4+ memory T cells could be used to predict good prognosis 36. Additionally, comparisons of immune scores revealed that high-risk patients possessed stronger immune functions than low-risk patients did (**Figure 7D**). Given the indispensable role of immune checkpoint blockade (ICB) in colon cancer therapy, we explored the discrepancy in the expression profiles of immune checkpoints between the two risk groups. Furthermore, the correlations between immune checkpoints and risk scores as well as risk genes are shown in **Figure 7L**.

Finally, we delved into the connections between gene mutations and different risk categories in order to gain a deeper understanding of the fundamental biological factors. First, we found that the proportion of mutations in the high-risk subgroup was greater than that in the low-risk subgroup. Moreover, we confirmed the top 20 mutated genes in different risk subgroups (**Figure 7M-N**). The mutation rates of APC, TP53, TTN, KRAS, PIK3CA, SYNE1, MUC16, FAT4 and RAY2 in different risk subgroups were greater than 20%. In addition, compared to patients with TP53 mutations, patients who carried wild-type TP53 had markedly lower risk scores (p<0.001, **Figure 7Q**), which could partly explain the high malignancy of patients in the high-risk group. Moreover, the tumor mutational burden (TMB) did not differ significantly between the two risk subgroups (**Figure 7O**), and the correlations between the two subgroups and the TMB were not significant (**Figure 7P**).

### Discrepancies in drug sensitivity, GSEA and GSVA between the two risk subgroups

**Figure 8:**
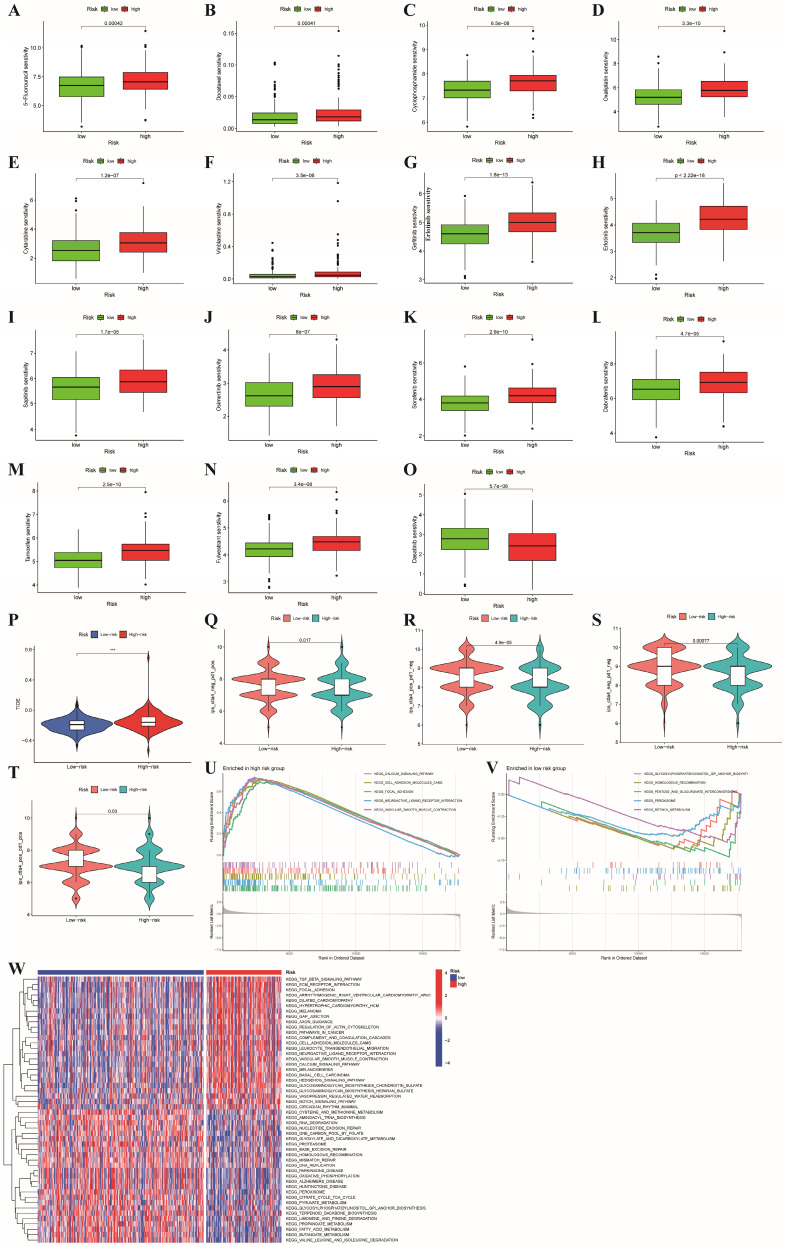
**Comparison of the two risk groups in routine drug sensitivity, molecular typing of immune escape and pathways.** (A-O) Correlations between the two risk subgroups and sensitivity to routine antitumor drugs. (P)The high-risk group had higher TIDE scores than the low-risk group; (Q-T) Comparison of immunophenoscore (IPS) between different risk subgroups; (U,V,W) GSEA and GSVA representing the different biological pathways between the high- and low-risk subgroups. P values are presented as follows: ***p < 0.001; **p < 0.01; *p < 0.05; ns: not significant;

For patients with unresectable CRC, the primary treatment is systemic therapy, including cytotoxic chemotherapy, targeted therapy, immunotherapy, biologic therapy, and their combinations, which may improve patient prognosis 37. First, we explored the associations between the risk subgroups and the effectiveness of chemotherapy and targeted therapies in patients with colon cancer based on the GDSC database with a total of 198 drugs using the OncoPredict algorithm. As shown in **Figure 8A-N**, we found that the low-risk subgroup had lower IC50 values for chemotherapy drugs (5-fluorouracil, docetaxel, cyclophosphamide, oxaliplatin, vinblastine and cytarabine) and targeted drugs (osimertinib, sapitinib, erlotinib, gefitinib, sorafenib, dabrafenib, tamoxifen and fulvestrant) (all p<0.001), which indicated that the sensitivities to commonly used therapeutic agents obviously differed between the two risk groups. Conversely, dabrafenib was more appropriate for the high-risk subgroup because of its lower IC50 value (p < 0.001, **Figure 8O**). Furthermore, to evaluate the status of immune escape in patients with colon cancer, we calculated the TIDE score to forecast the clinical efficacy of immunotherapy. Higher TIDE scores represent a greater potential for immune escape, illustrating that patients are unlikely to benefit from ICB 31. The high-risk subgroup had higher TIDE scores (**Figure 8P**). We further used TCIA to predict susceptibility to immunotherapy and revealed that the immunophenoscore (IPS) was significantly lower in the high-risk group compared to the low-risk group (all p<0.05, **Figure 8Q-T**). This finding illustrated that patients with high-risk characteristics may experience reduced effectiveness from immunotherapy.

GSEA and GSVA were applied to analyze the pathways enriched in different risk subgroups to discover molecular discrepancies. The GSEA results suggested that the genes in high-risk group were primarily involved in the calcium signaling pathway, cell adhesion molecules (CAMs), focal adhesion, neuroactive ligand receptor interaction, and vascular smooth muscle contraction (**Figure 8U**).

In the low-risk group, glycosylphosphatidylinositol (GPI) anchor biosynthesis, homologous recombination, pentose and glucuronate interconversions, and peroxisome and retinol metabolism were enriched (**Figure 8V**). Moreover, we applied GSVA to explore the discrepancies involved in KEGG pathway enrichment between the two subgroups, and the results are revealed in **Figure 8W**. These findings illustrate that tumor-associated signaling pathways may contribute to the distinct TMEs and drug sensitivities of different risk subgroups.

### PRELP inhibits the proliferation and migration of CRC cells

**Figure 9:**
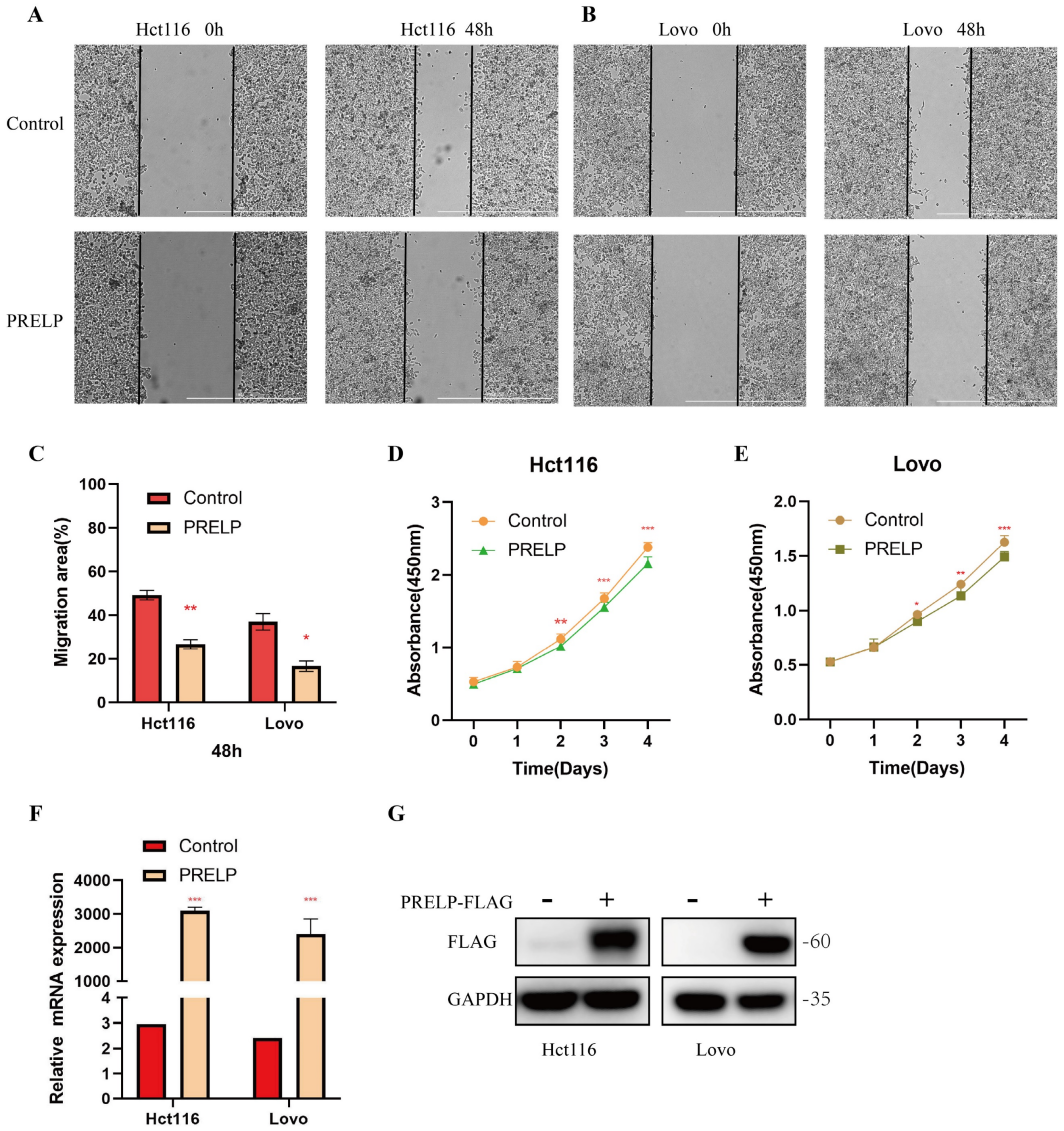
** PRELP inhibit the migration and proliferation of Hct116 and Lovo cells.** (A,B) PRELP inhibited the migration of Hct116 and Lovo cells based on scratch wound analysis. Scale bar: 1000μm. (C) Migration rate quantitative analysis in (A,B). (D,E) CCK-8 assay revealed that PRELP inhibited the proliferation of Hct116 and Lovo cells. (F,G) Detection of the expression of PRELP after exogenous transfection by qRT-PCR and WB analysis. *P < 0.05, **P < 0.01, ***P < 0.001 compared with the control group.

It has been shown that all of these risk genes, except PRELP, are related to colorectal cancer. Although PRELP has been found to be a tumor suppressor in hepatic cancer 38, bladder cancer 39 and retinoblastoma 40, its role in colorectal cancer is unclear. To further clarify its role in CRC, we conducted *in vitro* experiments. A scratch-wound healing assay was used to determine the effect of PRELP on the migration of CRC cells. PRELP significantly reduced the motility of Hct116 and Lovo cells and prevented cell migration (**Figure 9A-C**). CCK-8 analysis was performed on Hct116 and Lovo cells, and the results indicated that overexpression of PRELP resulted in a significant decrease in proliferation (**Figure 9D-E**). Besides, we conducted qRT-PCR and WB analysis to detection the expression of PRELP after exogenous transfection (**Figure 9F-G**). Above all, *in vitro* experiments uncovered that PRELP is a potential predictor of CRC.

## Discussion

Due to tumor heterogeneity, chemotherapy, targeted therapy and immunotherapy are currently ineffective for some patients with colon cancer 41. In this respect, new classifications and prognostic risk models for colon cancer are particularly needed to help promote individualized medicine. In the present study, we first identified ERGs and explored two molecular subtypes based on the screened ERGs that were markedly different in terms of prognosis and immune infiltration. Then, a prognostic risk model based on the DEGs between these two subtypes was identified by using 92 machine learning algorithm combinations. This model was further found to be a potential independent prognostic indicator with favorable predictive properties. Moreover, marked differences in immune infiltration and immune checkpoints between the two clusters and risk groups were detected. Furthermore, the relationship between the prognostic model with 12 risk genes and drug sensitivity was investigated, which may aid in the development of precise therapies for colon cancer.

It is predicted that genetic biomarkers and related diseases could be better understood with the advancement of interaction prediction research in various fields of computational biology. A recent report revealed that a new deep learning algorithm named GCNAT (graph convolutional network with graph attention network) could predict metabolites associated with disease and their potential associations 42. Additionally, novel deep learning predictive models, such as DMFGAM 43, were developed for predicting cardiotoxicity associated with hERG channel blockers. It has become equally important to study gene/protein signaling networks and establish theoretical models to understand regulatory mechanisms and find potential therapeutic targets. In this research, we established a prognostic risk model to predict the prognosis and therapy response of colon cancer using 92 algorithm combinations based on the integration of 10 machine learning algorithms, elucidated the different risk genes and then constructed a model influenced by ubiquitin modification.

Most of the twelve risk genes in the prognostic model have been implicated in tumor development, especially CRC. Wang et al 44 reported that DEPDC1 could promote the proliferation, invasion, and EMT of CRC via the regulation of zest12-mediated H3K27Me3. Additionally, DEPDC1 is associated with the sensitivity of CRC to 5-fluorouracil 45 and oxaliplatin 46. CDC25C is involved in regulating the G2/M phase and mediating DNA damage and repair, as well as a key part of tumorigenesis and tumor development 47-49. Recent research demonstrated that CDC25C was involved in the proliferation and cell cycle progression of CRC cells 50. PRELP plays a crucial role in regulating the EMT process and further inhibits the progression of retinoblastoma 40. In addition, CDCA2 can promote progression and may be related to poor prognosis and radioresistance in CRC 51. TIMP1 can accelerate tumor progression and metastasis by regulating the FAK-PI3K/AKT and MAPK pathways in colon cancer 52.

FSTL3 was found to be related to tumor invasion and metastasis in CRC by activating EMT 53, 54, and T-cell exhaustion and macrophage and fibroblast polarization 55. Furthermore, recent research has revealed that C2CD4A can interact with p53 and increase its ubiquitination and degradation to restrain the p53 signaling pathway in CRC 56. CXCR2, a type of chemokine receptor, has been reported to be expressed on CRC cells 57. Notably, the CXCR2 ligand CXCL2 can promote the development of colon cancer by binding to CXCR2 58, 59 and remodeling the TME 60-62. In CRC, the lncRNA ELFN1-AS1 can accelerate the proliferation, invasion and migration of tumors by targeting miR-4270 63, miR-191-5p 64, miR-1250 65, miR-4644 66 and miR-191-5p 67. In addition, the lncRNA ELFN1-AS1 can promote oxaliplatin resistance 68. GPX3 acts as both an oncogene and a tumor suppressor in different tumor types, suggesting that it plays a dichotomous role 69. In a meta-analysis of 17 eligible articles, Zhou et al 70 reported that GPX3 methylation is related to cancer and may be an important indicator for predicting lymph node metastasis. In colon cancer, Sarah and colleagues reported that HEYL inhibits the intravasation of metastatic CRC cells *in vivo*, hence negatively regulating metastasis formation 71. In colon cancer, the knockdown of SERPINE1 inhibits the EMT process, invasion and proliferation ability of cancer cells and increases apoptosis 72. Moreover, Kanto et al. discovered that the function of PRELP could be partially regulated by an HDAC inhibitor to suppress the development of bladder cancer. Given that PRELP play a role in tumor inhibition in hepatic cancer 38, bladder cancer 39 and retinoblastoma 40, the exact role of PRELP in CRC is not clear. Therefore, we further conducted an *in vitro* experiment and demonstrated that PRELP could inhibit the migration and proliferation of CRC cells. However, the potential molecular mechanisms by which risk genes influence the process of ubiquitination in colon cancer need further investigation.

As previously mentioned, considering the close connections between E3s and the TME, we used three immune analysis tools (CIBERSORT, ssGSEA, and ESTIMATE) to investigate the discrepancies in the immune response between the two risk subgroups based on 12 risk genes. Patients classified in the high-risk subgroup are very likely to experience poorer overall survival and a significantly modified tumor microenvironment. Increasing research has confirmed that ubiquitination plays a vital role in the immune response. Distinct modifications of proteins by ubiquitin in tumor or immune cells affect immune function to some extent 73, 74. Hence, detecting specific E3s-related biomarkers may help us better understand the immune mechanism of colon cancer. In the present research, the E3s-related genes applied for establishing the risk model to some extent was proved to be associated with tumor immunity and therapy. For instance, CDC25C has been proven to be degraded by the ubiquitin ligases BRCA1 75 and MDM2 76. It has been reported that CDC25C is associated with cell cycle arrest in macrophages 77 and T-cell leukemia/lymphoma cells 78. Additionally, in colorectal cancer cells with p53 mutations, CDC25C was shown to be involved in irinotecan-induced radiosensitization via the ATM/CHK/CDC25C/CDC2 pathway 79. A previous study revealed that SerpinE1 was negatively regulated by the E3 ligand RNF123 in aggressive glioblastoma tumors 80. Moreover, Serpine1 mRNA could accelerate the exclusion of CD8+ T cells from colon adenocarcinomas and confer mesenchymal characteristics to the cell 81. Additionally, SERPINE1 may serve as a biomarker of chemotherapy resistance in patients with soft tissue sarcoma 82. CDCA2 can act on SMAD-specific E3 ubiquitin protein ligase 1 and inhibit the degradation of ubiquitin-dependent Aurora kinase A (AURKA) to promote melanoma progression 83. Another study revealed that elevated CDCA2 expression was linked to the upregulation of immune checkpoints (PD-L1, PD-L2 and CTLA4) 84. Furthermore, CDCA2 could be involved in the growth inhibition of RCC cells by decitabine, which may function by suppressing p38/NF-κB signaling 85. This result indicates that the influence of ubiquitin modification on the expression of risk genes may be related to the extent of immune function and tumor progression. Thus, it revealed marked differences in sensitivity to routine chemotherapy, targeted therapy, and immunotherapy between the two risk subgroups, providing a new approach for combined immunotherapy and individualized treatments for colon cancer. In summary, these results illustrate that risk scores may be relevant to discrepancies in the immune response and could predict susceptibility to routine antitumor drugs as well as ICB.

Despite the potential clinical implications of our study, several limitations remain to be resolved. First, our study was a retrospective study, and the prognostic risk model needs to be validated with independent prospective cohorts. Second, although some studies have reported the relationship between these risk genes and tumors, this specific mechanism needs further experimental verification. Finally, the molecular mechanism of prognostic ERGs and their crosstalk with corresponding E3s in colon cancer are still unclear and need to be further investigated.

## Conclusions

In conclusion, we established a novel prognostic model using many bioinformatics and integrated machine learning algorithms and effectively validated it with internal and external colon cancer datasets. We demonstrated that this model is linked to the immune landscape and drug sensitivity in colon cancer. However, detailed research on ubiquitination and its corresponding target genes as well as downstream signaling pathways in colon cancer is still lacking. Moreover, the value of risk genes as potential drug targets deserves further investigation.

## Supplementary Materials

**Figure S1:** Flow chart of this research; **Figure S2:** Further validation of the OS-associated prognostic risk model for colon cancer; **Table S1:** E3s (n=240) obtained from previous literature; **Table S2:** Genes (62) obtained from univariate Cox analysis; **Table S3:** Machine learning algorithm combinations (92) and their corresponding C-indices and risk genes in all validation datasets. **Table S4:** Detailed clinic parameters of patients with colon cancer in TCGA dataset.

## Abbreviations

E3: E3 ubiquitin ligase
ERGs: E3-related genes
WGCNA: weighted gene coexpression network analysis
TCIA: The Cancer Immunome Atlas
GTEx: Genotype-Tissue Expression
TCGA: The Cancer Genome Atlas
IC50: half-maximal inhibitory concentration
CRC: colorectal cancer
TME: tumor microenvironment
GO: Gene Ontology
KEGG: Kyoto Encyclopedia of Genes and Genomes
GSEA: gene set enrichment analysis
TOM: topological overlap matrix
ROC: receiver operating characteristic
ssGSEA: single-sample gene set enrichment analysis
TIDE: Tumor Immune Dysfunction and Exclusion
CCK8: Cell Counting Kit-8
ICB: immune checkpoint blockade
TMB: tumor mutational burden
