# Supplementary material for: Bioinformatic Analyses and Integrated Machine Learning to Predict prognosis and therapeutic response Based on E3 Ligase-Related Genes in colon cancer
Source: J Cancer. 2024 Aug 19;15(16):5376–95. doi: 10.7150/jca.98723 (PMC11375543; doi:10.7150/jca.98723)
Supplement: Supplementary file 1 — Figure S1: Flow chart of this research; Figure S2: Further validation of the OS-associated prognostic risk model for colon cancer; Table S1: E3s (n=240) obtained from previous literature; Table S2: Genes (62) obtained from univariate Cox analysis; Table S3: Machine learning algorithm combinations (92) and their corresponding C-indices and risk genes in all validation datasets. Table S4: Detailed clinic parameters of patients with colon cancer in TCGA dataset. [file jcav15p5376s1.zip › Figure 1S-2S.pdf]

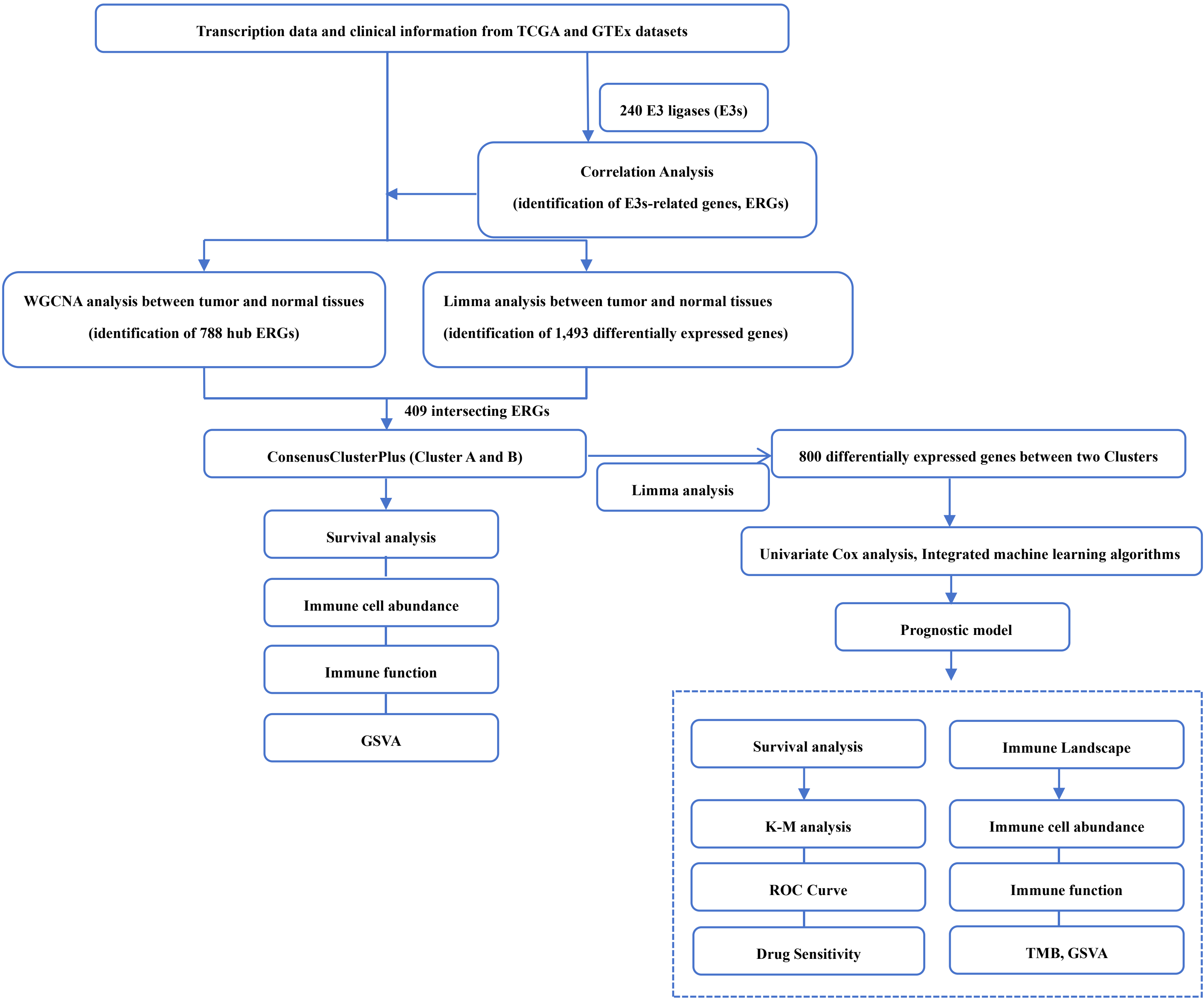

Figure S1: Flow chart of this research.

First, we applied Spearman correlation analysis and identified 6,268 ERGs (E3-related genes) in colon cancer samples based on 240 E3s (E3 ubiquitin ligases) from the TCGA and GTEx datasets. Then, WGCNA and Limma analysis were used to identify meaningful ERGs between tumor and normal tissues respectively and 409 intersecting genes were identified. Based on the overlapping ERGs evaluated, we identified distinct prognostic subtypes using consensus clustering and revealed that the best number of clusters was two; these clusters were denominated A and B. We performed survival analysis, immune cell abundance,

immune function and GSVA to clarify the differences between the two clusters. To further study the potential mechanisms of these two tumor clusters, we identified 800 DEGs (differentially expressed genes) between clusters A and B via Limma analysis. Moreover, univariate Cox regression analysis and integrated machine learning algorithms were used to construct the prognostic model. We also evaluated the E3-related risk model in terms of survival analysis, drug sensitivity and other factors. The correlation between the immune response and the prognostic model was also assessed.

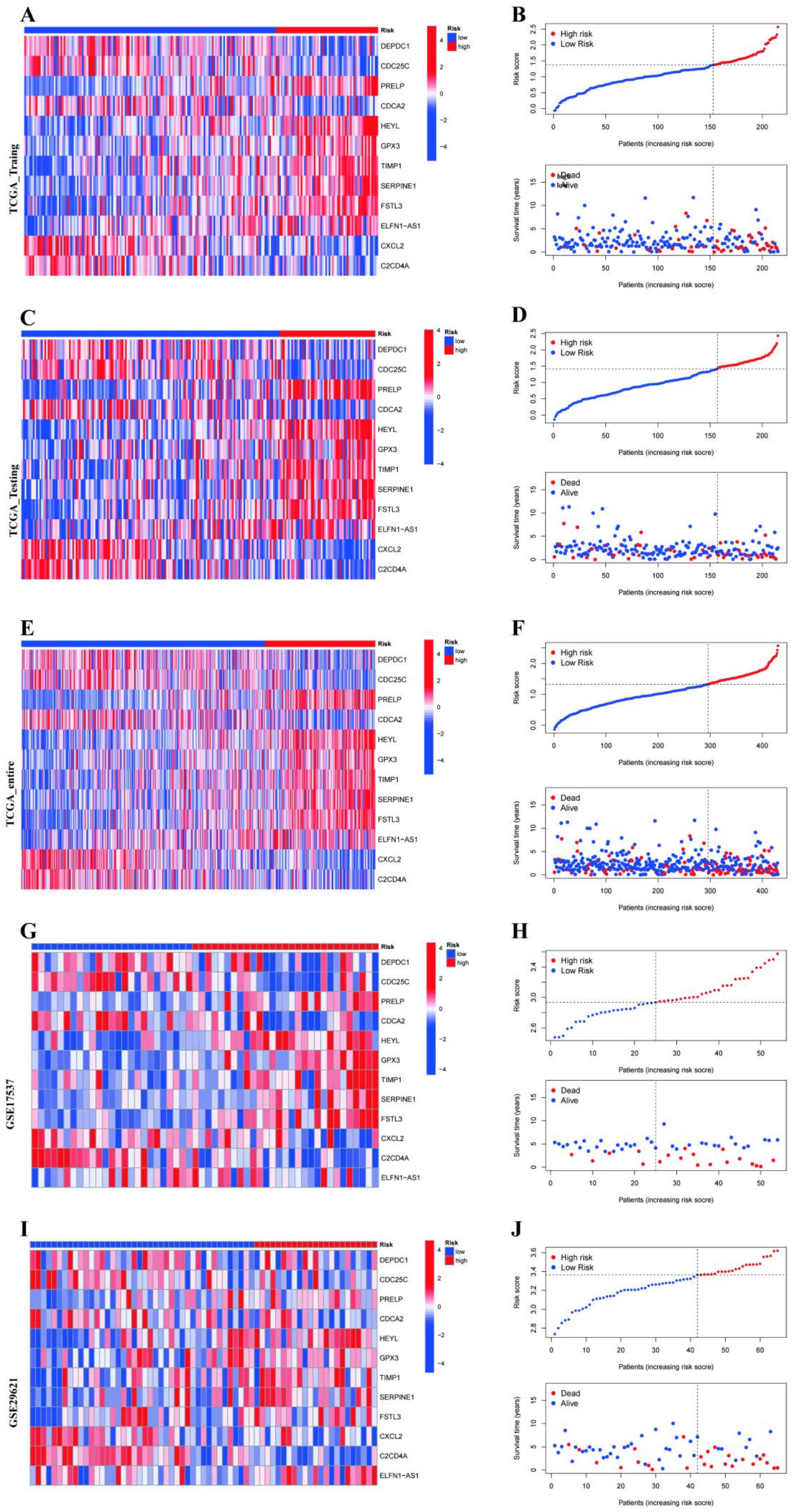

A
B
C
D
E
F
G
H
I
J
TCGA_Traing
TCGA_Testing
TCGA_entire
GSE17537
GSE29621
Risk
low
high
DEPDC1
CDC25C
PRELP
CDCA2
HEYL
GPX3
TIMP1
SERPINE1
FSTL3
ELFN1−AS1
CXCL2
C2CD4A
High risk
Low Risk
Risk score
Patients (increasing risk socre)
Dead
Alive
Survival time (years)

Figure S2: Further validation of the OS-associated prognostic risk model for colon cancer. (A, B) The distributions of the prognostic model, survival status, and expression profile of risk genes in the training dataset. The distributions of the prognostic model, survival status, and expression profile of risk genes in the TCGA Testing dataset (C,D), TCGA entire dataset (E,F),GSE17537(G,H) and GSE29621(I,J).
